# The leafhopper genus *Multiproductus* Xing, Dai & Li in China (Hemiptera, Cicadellidae, Deltocephalinae, Paralimnini), with description of one new species

**DOI:** 10.3897/zookeys.369.6614

**Published:** 2014-01-13

**Authors:** Ji-Chun Xing, Zi Zhong Li

**Affiliations:** 1 Institute of Entomology, Guizhou University; Guizhou Key Laboratory for Plant Pest Management of Mountainous Region, Guizhou University, Guiyang, Guizhou Province, 550025, China.

**Keywords:** Homoptera, morphology, taxonomy, distribution

## Abstract

General characteristics of *Multiproductus* and a new species *Multiproductus complantus* **sp. n.** are described and illustrated. A key is given to distinguish all species of the genus.

## Introduction

The Oriental leafhopper genus *Multiproductus* belonging to Paralimnini of Deltocephalinae (Hemiptera: Cicadellidae), was established by Xing et al. (2011) for a single species, *Multiproductus ramosus* Xing, Dai & Li, 2011, from China. Here we describe and illustrate a second new species: *Multiproductus complantus* Xing & Li,sp. n. from Guizhou Province, China. A key is given to distinguish all species of the genus.

## Material and methods

Specimens were collected by sweeping net. Dry specimens were used for the description and illustration. External morphology was observed under a stereoscopic microscope and characters were measured with an ocular micrometer. The genital segments of the examined specimens were macerated in 10% NaOH and drawn from preparations in glycerin jelly using a Leica MZ 12.5 stereomicroscope. Illustrations were scanned with Canon CanoScan LiDE 200 and imported into Adobe Photoshop CS3 for labeling and plate composition.

Terminology of morphological and genital characters follow Xing et al. (2011) and Rakitov (1998). The examined specimens and type specimens of the new species are deposited in the Institute of Entomology, Guizhou University, Guiyang, China (GUGC).

## Taxonomy

### Multiproductus

Xing, Dai & Li

http://species-id.net/wiki/Multiproductus

Multiproductus Xing, Dai & Li, 2011: 65.

#### Type species.

*Multiproductus ramosus* Xing, Dai & Li, 2011.

#### Remarks.

For the relationship and diagnosis of *Multiproductus* Xing, Dai & Li, 2011 see Xing et al. (2011: 65). *Multiproductus* is distributed in China (Oriental Regions). This genus is especially well differentiated from other genera of Paralimnini in Deltocephalinae by the unique forewing with outer subapical cell extended to costal margin, branches of vein R recurved distally, resulting in fifth (outer) apical cell. The two species of this genus are very similar in appearance. This situation is similar to the paralimnine genus *Paralaevicephalus* wherein all species are essentially the same in external appearance and several different species can be collected in the same location.

#### Distribution.

China (Guizhou, Yunnan, Hainan).

#### Key to species (♂) of *Multiproductus*

**Table:** 

1	Aedeagal shaft with three pairs of lateral preapical processes; apical process of style narrow, tubular shape (see Xing et al. 2011: Figs 9, 10, 13)	*Multiproductus ramosus*
–	Aedeagal shaft with two pairs of lateral preapical processes; apical process of style wide and flat, sword shape (Figs 9, 10, 13)	*Multiproductus complantus* sp. n.

### Taxonomy of species

#### Multiproductus ramosus

Xing, Dai & Li, 2011

http://species-id.net/wiki/Multiproductus_ramosus

Multiproductus ramosus Xing, Dai & Li, 2011: 66–67, figs 1–15.

##### Material examined.

China: 1♂ (Holotype), Guizhou Prov., Guanling County, Huajiang, 16 August 2009, coll. Jichun Xing; 2♂♂4♀♀, Guizhou Prov., Guanling County, Huajiang, 16 August 2009, coll. Jichun Xing; 1♂, Guizhou Prov., Ziyun County, Baishiyan, 28 July 2008, coll. Jichun Xing; 1♀, Guizhou Prov., Yanhe County, Mayanghe, 10 June 2007, coll. Jichun Xing; 1♀, Yunnan Prov., Menghai County, Mannong, 24 July 2008, coll. Yuehua Song; 1♂, Yunnan Prov., Baoshan City, Baihualing, 7 May 2010, coll. Yanli Zheng; 1♂1♀, Hainan Prov., Bawangling, 16 April 2013, coll. Jichun Xing.

##### Distribution.

China (Guizhou, Yunnan, Hainan).

#### Multiproductus complantus

Xing & Li sp. n.

http://zoobank.org/28A036CA-70C0-48B7-8410-D0D405913F75

http://species-id.net/wiki/Multiproductus_complantus

Figs 1 –13

##### Description.

Yellowish-brown species, with light veins on forewings. Crown yellowish brown with four dark brown marks on anterior margin and orange–yellow longitudinal band midway between midline and eye extending to posterior margin of pronotum. Eyes black, fairly large. Ocelli pale yellow. Face black, frontoclypeus with yellowish brown transverse stripes on both sides. Forewings pale yellow. Hind macropterous. Legs marked with brown.

Head slightly wider than greatest width of pronotum. Vertex with fore margin produced triangularly, median length longer than width between eyes. Ocelli on anterior margin, separated from corresponding eye by approximately their own diameter. Frontoclypeus distinctly longer than wide, anteclypeus slightly narrowed apically. Antennae arising near lower corner of eye. Pronotum with anterior margin strongly and roundly produced, posterior margin slightly concave. Scutellum triangular, slightly shorter than pronotum, with transverse suture curved and depressed. Forewing with outer subapical cell extended to costal margin, branches of vein R recurved distally, resulting in fifth (outer) apical cell, and veins of clavus appear to extend to the claval suture, 4 times as long as wide, appendix present. Hind wings with three apical cells and two anteapical cells. Profemur with 2 dorsoapical setae. Hind femur apical setal formula 2+2+1. Hind tibia flattened and nearly straight, with PD setae very long, several supernumeral setae present between AD and AV rows; AD row with somewhat thin setae between very thick macrosetae. Metabasitarsomere with three platellae and two setae on apical transverse row; plantar surface with one row of five stout setae at middle and one row of four stout setae at lateral margin.

*Male genitalia*. Male pygofer side elongate with many large setae medially; without processes (Fig. 5). Valve subtriangular with anterior margin produced and posterior margin strongly produced medially (Fig. 6). Subgenital plate wide, with uniseriate row of macrosetae along lateral margin, internal appendage short and mucronate (Figs 7, 8). Aedeagal shaft elongate and sinuate; with two pairs of lateral preapical processes, proximal pair with two small spines; gonopore subapical on ventral surface (Figs 9, 10). Connective loop–shaped with arms fused apically; stem present, articulated with the aedeagus (Figs 11, 12). Apical process of style wide and flat, sword shape (Fig. 13).

**Figures 1–4.**
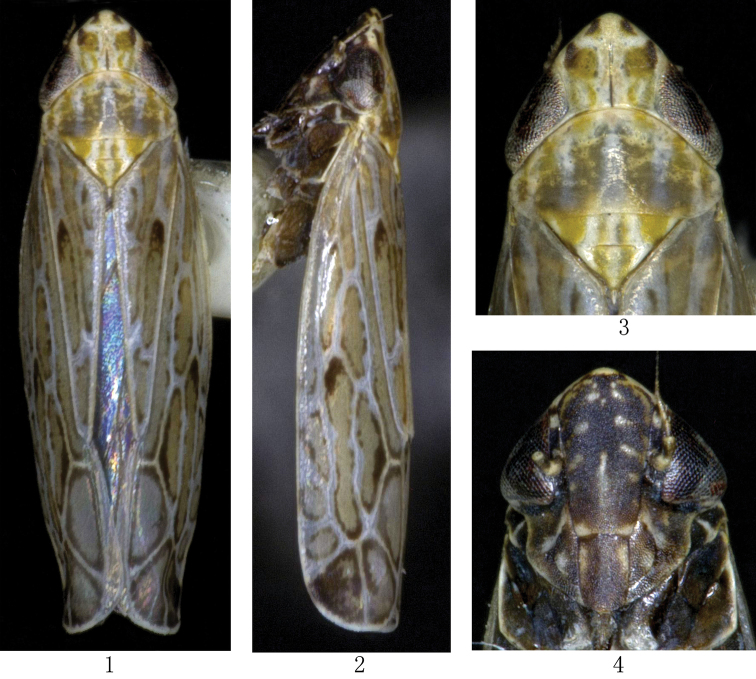
*Multiproductus complantus* sp. n. **1** ♂, dorsal view **2** ♂, lateral view **3** Head and thorax,dorsal view **4** ♂,face.

**Figures 5–13.**
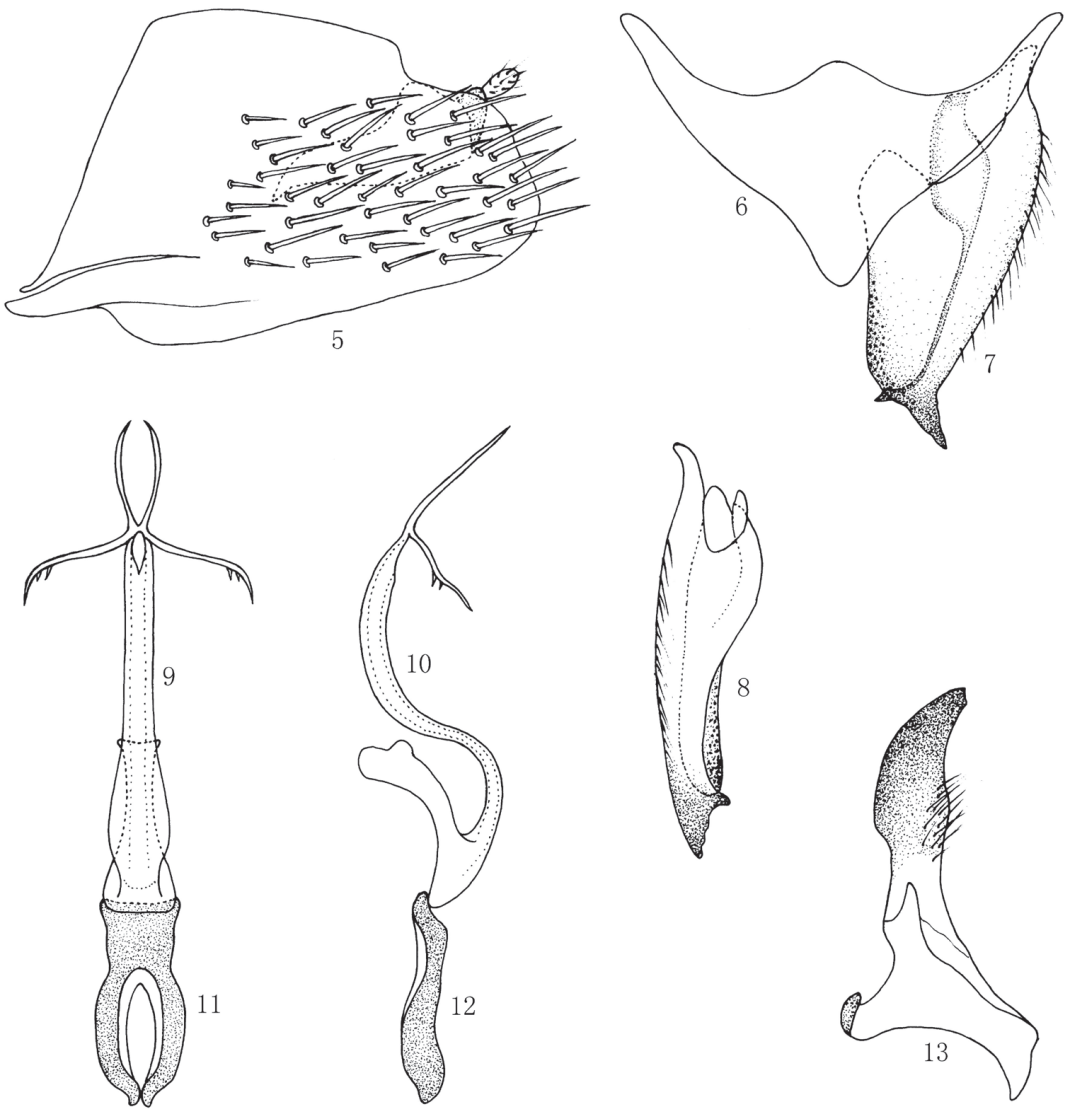
*Multiproductus complantus* sp. n. **5** Male pygofer side, lateral view **6** Valve, ventral view **7** Subgenital plate, ventral view **8** Subgenital plate, lateral view **9** Aedeagus, ventral view **10** Aedeagus, lateral view **11** Connective, ventral view **12** Connective, lateral view **13** Style, dorsal view.

##### Measurement.

Length (including tegmen): ♂, 3.0 mm.

##### Host.

Grasses.

##### Type material.

Holotype ♂, China: Guizhou Prov., Ziyun County, Baishiyan, Kazha, 2 October 2013, coll. Jichun Xing (GUGC).

##### Diagnosis.

This new species is similar to *Multiproductus ramosus* Xing, Dai & Li, 2011 in appearance, but can be distinguished from the latter by the aedeagal shaft with two pairs of lateral preapical processes, the apical process of style wide and flat, sword shape, and the valve subtriangular and subgenital plate wide.

##### Etymology.

The species name is derived from the Latin word “*complantus*”, referring to the apical process of style wide and flat.

## Supplementary Material

XML Treatment for Multiproductus

XML Treatment for Multiproductus ramosus

XML Treatment for Multiproductus complantus
